# Correspondence

**Published:** 1881-11

**Authors:** 


### Correspondence.

VIENNA, October 15, 1881.

*Editors of the Journal:*—Perhaps some of the observations ot a medical student abroad may have interest to others beyond himself, and it is with this belief that the present letter is sent. It is quite common for those intending to study in German universities to spend a few months previous in some North German town for the purpose of perfecting themselves in the language. Generally some Brunswick city is chosen, for the finest German is spoken in this province—either Hannover or Brunswick. Therefore I went to Göttingen, and to a physician I found it afforded also advantages for medical study. There are two or three hospitals and a large provincial insane asylum, and we could visit these whenever we pleased. The "theatrum anatomicum" has a celebrated anatomist at its head—Henle—and his anatomical museum was a pleasant place to pass away leisure hours. In it is the well-known collection of skulls by Blumenbach, comprising several hundred, and even in a cursory examination one noticed the square-skulled English, backward-sloping Chinese skulls, protruding-jawed Irish, narrow-jawed Russian, prominent Swedish cheek bones, narrow-skulled Danes, round, thick African skulls, and the well-shaped ancient Greek. The Chemical Laboratory seems to draw many Americans to Göttingen; Prof. Hübner is the chief chemist. Some twelve young Americans are making a specialty of this science under him. The university has Prof. König in the department of surgery. The insane asylum contains some three hundred and fifty patients, mostly chronic. The principle of treatment is merely to make the asylum like a home to them. They work, have concerts, balls, go to church, etc., just as if sane and at home. I noticed there and in many other similar institutions later, that the methods of ventilation and heating are far from perfect. They often use their old-fashioned porcelain stoves in the wards. The Göttingen surgeons must become exceedingly expert in the use of sutures, if one may judge by the numberless scarred faces of the students. One would think the scars average nearly seven to each of the 1,000 students in the town. Duelling is merely a sport among them, a pleasant pastime, but is also often resorted to, to satisfy their imaginary honor. The loss of a piece of nose or ear, a slash through the cheek, chin or forehead, is generally sufficient to satisfy it; but while I was there, one had his brain thrust through by a sword and another was shot fatally through the lung.

I remember once reading somewhere that difficult labor is very much more common among the German women than those of other nations. One would not wonder at this when he sees the amount of hard work the woman performs in Germany. Always in my numerous walks into the country I would see them along the roads carrying on their shoulders a hundred-weight of market produce, or hoeing in the fields, mowing in the meadows, and once on the Elbe river loading a boat with coal, and quite often carrying mortar and brick up some lofty building. Apparently they are very healthy and strong, but we notice that they are soon old. The heavy labor makes closer-set pelves, and very unyielding ones, and this may be the reason for a great per cent. of instrumental interference in childbirth. The sewer system of most of the German towns of this size, at least of a dozen or so I visited, are very defective. The sewer is a superficial gutter down which all sorts of fluids flow under the hot sun, while the solids are scraped together and carted away. Therefore it is that in Göttingen, within its old walls, the summer brings with it a fever of a typhoid type. Students can always in Göttingen find pleasant families outside the wall in which to have practice in German conversation. I spent part of July and all of August in this quiet, studious village, and then betook myself to the Harz mountains and Thüringia for a two-weeks' tour a-foot. There are several things a medical student will notice in such a tramp. First, I passed one day through the village of Lerbach in the Oberharz, whose one long, straggling street in a narrow valley showed me so many examples of goitre and cretinism. I had hardly anticipated these before reaching Switzerland next year. They sat stupidly—mostly dwarfed children—before their doors and generally held out their hands for pennies. This village is the one most populated with these deformed beings. It is situated in a rich iron region, the great mines of Clausthal being but a few miles off, and the hillsides here are covered with iron ore. It is also a limestone region, and the water percolating through this and running over it, may, as has been said, be a cause of the disease in those who drink of it from their infancy. Nothing further in the Harz mountains was professionally interesting, if I may except the baths at the much-frequented summer resort Harzburg in the Vorharz, and these not so much as the beautiful village itself, which I thought would be an enchanted land to some one whose nerves were all unstrung or who was invalided by overwork. What beneficial influence on such would not the green meadows have, the fir trees, the beech walks, the mountain air, the baths, the music, the Ladanthal, the Ockerthal, the Ilse river, and a trip to the Brocken! Nordhausen, which I soon after visited, is noted for fine chemical works, and especially for the manufacture of sulphuric acid, and works of this character I also made it a point to visit. A few days later I came to Gotha to see—its Crematorium. In this, of course, I had a very deep interest. The Crematorium, built and owned by the city of Gotha (be it said to its honor and credit), is a one-story building of the fine old Roman architecture. It consists of two wings, united by a columned corridor, which is its Columbarium. In the left wing are the doctor's office, a *post-mortem* examination room, and a large hall in which the dead may lie in state. In the right wing are the office of the priests, the stairway descending to the furnace rooms, and the handsome hall where funeral services are performed. In the centre of the funeral hall is a platform for the coffin, over which is spread a pall. While the service is going on, the coffin sinks noiselessly and unseen, the pall remaining, to the furnace beneath; the body is placed on an iron slab, which moves into the white-hot brick oven, and in a short time is consumed—not by flames of fuel, however, but by burning gas; by the end of the service the removal of the pall reveals the urn containing the ashes of the dead one. The apparatus is that of Siement of Dresden, and the Crematorium was constructed under his direction. Fifty-eight have already been cremated, and tasteful urns containing their ashes stand upon pedestals in the Columbarium. The building is situated in a cemetery newly laid out by the city. To a medical man the contrast could not but be great between the poetic, Grecian urns and their wreaths of flowers, and the graves of others in the field into which and whose impurities his thoughts could not fail to dive.

Three weeks in Leipzig afforded me many opportunities to visit the institutions belonging to the medical department of its university—the anatomical laboratory of His and Braune, the pathological of Cohnheim, and the physiological of Ludwig, and the fine pavilion general hospital with beds for 600 patients. Many of the professors were taking their vacations, but I had the advantage during my stay there of the interesting chirurgical poliklinik of Prof. Schmidt.

A week in Berlin did not profit me much—medically. It was just a few weeks before the beginning of the Semester, and beyond a hasty visit to the hospitals and to the magnificent anatomical museum, I did nothing for the increase of my professional knowledge. At Dresden there was little to see except a fair hospital. In both Berlin and Dresden the arts occupied the best portion of my time and thoughts. From Dresden I made a pleasant tour through the Saxon Snitzel land to the Bohemian capital Prague. After the historical associations of the quaint old city, its Hradschin, Jews' Quarter and broad Moldau river, came a visit to the General Hospital and the Lying-in Asylum, and the last is what Prague can well boast of. The first assistant was good enough to show me everything in it and to explain everything satisfactorily. It consists of a large main building three stories in height, a court and three wings on each side. The number of births average four daily, and the unusual opportunity is given students, of a residence in the hospital for a very small sum of money indeed, and a bell startles him up at every birth about to take place.

But now I am in Vienna and established for many months. A course of hard study begins two days from now. Already I have attended several of the surgical clinics of Prof. Albert and the gynecological of Prof. Spaeth. I have found a room within two minutes of the Allgemeine Krankenhaus, where most of the clinics are held. Some idea of the amount of material here—dead and living—may be gained from the statement that last year 24,000 patients were treated in this hospital alone, of whom 13 per cent. died. But this hospital is not the only one, though the chief. There are thirty others. One can see cases all day long and every day in the week. I must bring this letter to a close. I fear it does not contain much of medical importance; but I hope later to have gathered material in the way of cases and descriptions of professors which will prove of more interest, and from which I can fashion a letter or two for the JOURNAL.

Sincerely, F. P.

